# Problematic Social Media Use Among Italian Midadolescents: Protocol and Rationale of the SMART Project

**DOI:** 10.2196/58739

**Published:** 2024-09-09

**Authors:** Valeria Donisi, Laura Salerno, Elisa Delvecchio, Agostino Brugnera

**Affiliations:** 1 Department of Neuroscience, Biomedicine and Movement Science Section of Clinical Psychology University of Verona Verona Italy; 2 Department of Psychology, Educational Science and Human Movement University of Palermo Palermo Italy; 3 Department of Philosophy, Social Sciences and Education University of Perugia Perugia Italy; 4 Department of Human & Social Sciences University of Bergamo Bergamo Italy

**Keywords:** adolescents, social media, problematic social media use, psychological distress, wellbeing promotion, eHealth interventions, co-creation, qualitative research, biomarkers

## Abstract

**Background:**

Social media (SM) use constitutes a large portion of midadolescents’ daily lives as a way of peer interaction. A significant percentage of adolescents experience intense or problematic social media use (PSMU), an etiologically complex behavior potentially associated with psychological distress. To date, studies longitudinally testing for risk or protective factors of PSMU, and collecting qualitative data are still scarce among midadolescents. Self-help interventions specifically targeting PSMU in this population and involving midadolescents in co-creation are needed.

**Objective:**

The 2-year SMART multicenter project aims to (1) advance knowledge on PSMU; (2) co-design an unguided self-help app for promoting awareness and functional SM use; and (3) test feasibility and provide preliminary findings on its effectiveness to further improve and adapt the app.

**Methods:**

The SMART project is organized in 3 phases: phase 1 will focus on knowledge advancement on PSMU and its risk and protective factors using a longitudinal design; phase 2 will explore adolescents perspectives using qualitative approach and will co-design an unguided self-help app for reducing PSMU, which will be evaluated and adapted in phase 3. Around 1500 midadolescents (aged 14-18 years) will be recruited in northern, central, and southern Italy to investigate the potential intra- and interpersonal psychological risk and protective factors for PSMU and define specific PSMU profiles and test for its association with psychological distress. Subjective (self-report) PSMU’s psychosocial risk or protective factors will be assessed at 3 different time points and Ecological Momentary Assessment (EMA) will be used. Moreover, focus groups will be performed in a subsample of midadolescents to collect the adolescents’ unique point of view on PSMU and experiences with SM. Those previous results will inform the self-help app, which will be co-designed through working groups with adolescents. Subsequently, the SMART app will be deployed and adapted, after testing its feasibility and potential effectiveness in a pilot study.

**Results:**

The project is funded by the Italian Ministry of University and Research as part of a national grant (PRIN, “Progetti di Rilevante Interesse Nazionale”). The research team received an official notice of research funding approval in July 2023 (Project Code 2022LC4FT7). The project was preregistered on Open Science Framework, while the ethics approval was obtained in November 2023. We started the enrollments in December 2023, with the final follow-up data to be collected within May 2025.

**Conclusions:**

The innovative aspects of the SMART project will deepen the conceptualization of PSMU and of its biopsychosocial antecedents among midadolescents, with relevant scientific, technological, and socioeconomic impacts. The advancement of knowledge and the developed self-help app for PSMU will timely respond to midadolescents’ increased loneliness and psychological burden due to COVID-19 pandemic and humanitarian crisis.

**Trial Registration:**

OSF Registries; https://osf.io/2ucnk/

**International Registered Report Identifier (IRRID):**

DERR1-10.2196/58739

## Introduction

### Background

According to the World Health Organization, one-sixth of the global population is composed of adolescents, and this number is expected to rise through 2050 [1]. Unfortunately, 1 in 7 adolescents experience mental health conditions, which remain largely unrecognized and untreated. Also, given the widespread use and pervasiveness of new technologies among adolescents, it is not surprising that 60.43% of Italian adolescents report an intense or a problematic social media (SM) use (PSMU) [2] and, although to our knowledge, no estimations regarding the direct or indirect financial cost or societal burden of PSMU are currently available, it has been hypothesized that PSMU may have relevant costs both for individuals (eg, mental and physical health, social functioning) and societies (eg, direct health care costs and indirect costs following productivity losses).

SM is made up of various digital user-driven platforms (eg, Facebook, TikTok, Instagram) that facilitate connectivity and engagement between individuals as well as allow to create and share user-generated content [3]. PSMU is considered an etiologically complex behavior associated with psychological distress, although this relation is far from being completely understood [4] and the conceptualization of PSMU is still debated. To date, the absence of a commonly accepted definition could be linked to the rapidly changing nature of SM as well as to the inconsistent results obtained by prior research on PSMU among adolescents [5]. Technology and SM are an integral part of the daily life of today’s teenagers, who use them to create a virtual space to satisfy their needs for communication, social contact, belonging, comparison, and self-realization. However, some scholars defined PSMU as an “addictive-like” behavior (eg, associated with salience, tolerance, withdrawal, and relapse) [6], hierarchically arranged (ie, with different levels of excessive or disordered use) and whose main characteristic is a lack of self-regulation in one’s own SM use [4].

Nonetheless, the adoption of an addiction paradigm to describe PSMU has been criticized [7].

Further, recent findings have highlighted how PSMU cannot be defined solely by the so-called screen-time (“how much”), but rather by “how” the SM is used [8]. More specifically, a high intensity of screen time and SM use may be a normative adolescent behavior that does not necessarily interfere with life domains relevant to adolescents’ mental health (eg, offline socializing with friends or family) [2]. Core aspects of the way in which SM are used are active versus passive use [9], social support [10], fear of missing out [11], and types of activities individuals engaged in when using SM [12]. Yang et al [13] proposed an integrative model in which the previous multiple dimensions of SM use are considered. The Multidimensional Model of Social Media Use [13] suggests that individual platforms differ by activities (eg, interactive or directed communication, active posting to an unspecified audience, passive browsing), motives for use (eg, enhancement or compensation), and communication partners (eg, strong or weak ties) and that each dimension is associated with better or poorer well-being among youth. However, despite these differences in SM platform activities and interaction partners, surprisingly research usually focuses on overall or generic SM use and few studies have examined how adolescent characteristics may be more relevant to some platforms than others [14,15].

Previous research has also investigated several potential intra- and interpersonal psychological predictors of PSMU, clearly evidencing its multifactorial and complex etiology [16]. Among others, secure attachment [17], self-esteem [18], self-control [19], emotion regulation [20], life satisfaction [21], and parental variables (such as low levels of parents’ own SM use and parental phubbing) [22] are some of the established protective factors for PSMU. Despite such efforts, the mechanisms underlying the development and maintenance of PSMU are still underinvestigated [4,23] due to the lack of (1) well-performed longitudinal studies designed to test for a temporal precedence of specific risk and protective factors for PSMU, (2) qualitative studies collecting adolescents’ unique perspectives and needs, and (3) psychophysiological studies on PSMU. Indeed, 2 biomarkers of psychopathological risk, namely a lower parasympathetic activity over heart at rest [24], and the presence of specific polymorphisms for serotonin gene transporter 5-HT1AR and 5-HT2AR [25], may be associated with increased odds of PSMU among adolescents.

Finally, there is a call for effective prevention or early intervention programs which are tailored to the substantial risk and protective factors for PSMU to promote awareness of PSMU phenomenon and functional SM use and to overcome less effective abstinence-based protocols [26]. Smartphone apps could be an innovative way to reach young people. There is increasing evidence that unguided mobile self-help apps and web-based interventions offer a powerful solution for promoting public health and life skills interventions [27,28], especially among young digital natives [29,30]. The use and the engagement of such self-help apps may also increase thanks to a co-design process that involves end users (ie, midadolescents) [27,30,31].

### Objectives of the SMART Project

The 2-year project “problematic Social Media use among Italian mid-Adolescents: from the identification of Risk/proTective factors to the co-design and evaluation of a self-help app” (namely “SMART project”) aims to: (1) advance knowledge on PSMU and its risk and protective factors using a longitudinal design; (2) exploring adolescents unique perspectives on PSMU and co-design an unguided self-help app for promoting awareness and functional SM use; and (3) test feasibility and provide preliminary findings on its effectiveness to further improve and adapt the app.

As for the first aim, we hypothesize that intrapersonal (ie, higher difficulties in emotion regulation, negative affect, adjustment difficulties, and fear of missing out, as well as lower self-esteem, self-control, and satisfaction with life) and interpersonal (ie, attachment insecurity, higher preference for online social interactions, and parental phubbing and parents’ dysfunctional SM use) risk factors will predict higher adolescents’ PSMU (primary outcome), as well as higher emotional distress and passive SM use (secondary outcomes).

As for the second aim, the research questions that guide the study are: what are the adolescents’ experiences and perspectives on SM and PSMU, the SM impact, and the possible strategies for promoting positive SM use? As for the app, we hypothesize to include several interactive modules, including an informative and awareness section and a psychoeducational section, to promote both (1) emotional awareness and skills, and (2) adolescents’ personal and interpersonal resources, so to prevent PSMU and promote a functional SM use.

As for the last aim, we hypothesize a good feasibility for the SMART app, and that greater app use will be associated with an increase in functional SM use.

## Methods

### Study Design

The SMART project is organized in 3 consequential phases, each including different integrated actions: phase 1—prospective longitudinal study with repeated-measures design [32] for the advancement of knowledge on PSMU through quantitative approaches; phase 2—qualitative focus group study for exploring adolescents’ perspectives on PSMU and functional use of SM and co-design approach for developing the unguided self-help SMART app; phase 3—pilot study to evaluate feasibility and signal of effectiveness of the intervention throughout the self-help app.

**Figure 1:**
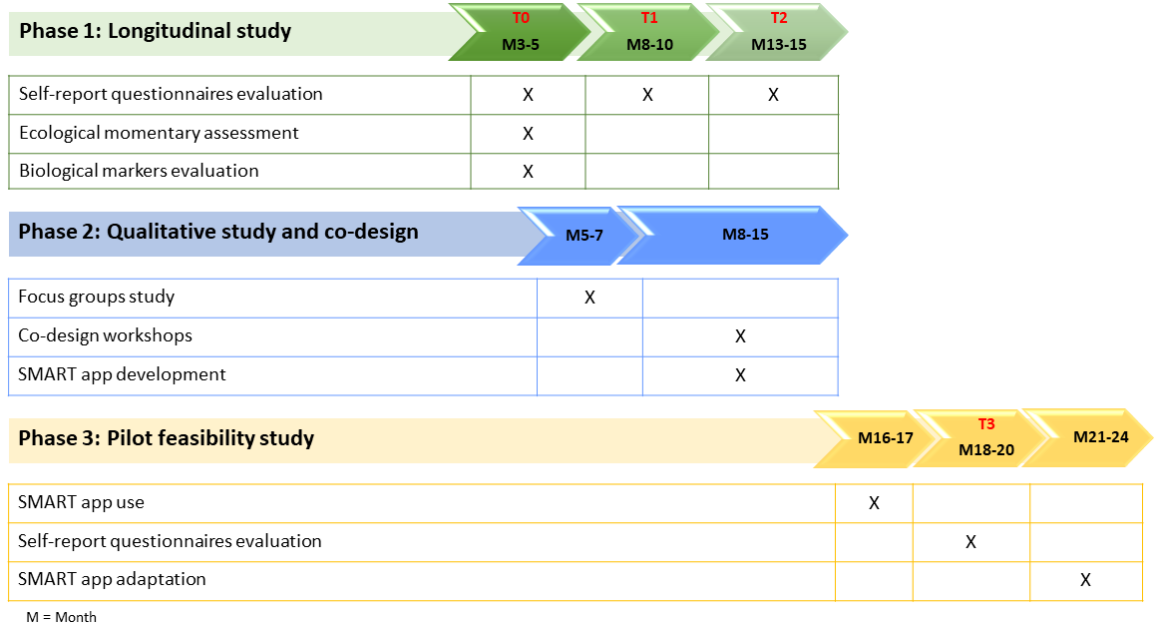
SMART project: General overview of the 3 phases.

The project is conducted by researchers and clinical psychologists in four Italian Universities (University of Verona, University of Palermo, University of Perugia, and University of Bergamo), thus including a large population of midadolescents in diverse regions of north, central, and south of Italy (see Figure 1 for an overview of the project phases and Table 1 for a synthetic description of general objectives, research aims, and methods of each phase).

**Table 1:** General description of the 4 study phases: objectives, research aims, and main methodologies.

Phase	General objectives	Research aims	Main methodologies
Phase 1: Longitudinal prospective study	Improving the conceptualization of PSMU^a^ Longitudinally identifying the intra- and interpersonal risk, protective and maintenance factors of PSMU Longitudinally analyzing the associations between PSMU and psychological distress Exploring the presence of specific profiles of PSMU among adolescents Informing the development of SMART self-help app	Which are the main risk or protective factors of PSMU among adolescents? What are the longitudinal associations between PSMU and psychological distress? Is PSMU predicted by a higher distress, or is it a change in distress for that specific participant to predict it? Are there specific sub-groups of PSMU? If yes, what characterizes them?	Prospective longitudinal study with repeated-measures design (3 waves) using self-report questionnaires Collection of biological markers EMA^b^
Phase 2: Qualitative study and co-design of the SMART app	Exploring adolescents’ experiences, examples and perspectives on PSMU and functional use of SM^c^ Informing the development of SMART self-help app Co-designing the beta version of a self-help app aiming to promote awareness and functional SM use	Which are the adolescents’ experiences and perspectives on PSMU and functional use of SM? Which are the adolescents' opinions and perspectives on what is useful to promote awareness and functional SM use? Which are the adolescents’ perspectives, suggestions, and preferences on an app to promote functional SM use?	Focus groups Thematic analysis of the transcripts to identify and analyze patterns in qualitative data Co-design working groups with the adolescents involved
Phase 3: Feasibility and pilot study	Investigating the feasibility and acceptability of the SMART self-help app Preliminary evaluating its effectiveness Incorporating user feedback and study results during the app development	Which are the perspectives of adolescents on the SMART self-help app feasibility and acceptability? Is there preliminary evidence that the SMART self-help app is effective?	Single-arm pilot feasibility study to explore feasibility and preliminary data on effectiveness (pre-post evaluation)

^a^PSMU: problematic social media use.

^b^EMA: ecological momentary assessment.

^c^SM: social media.

### Participant Recruitment and Setting

The target group (adolescents aged 14-18 years) will be actively involved in the various actions of each phase. Participants will be recruited from schools (ie, secondary school), in the north, central, and south Italy. Each research team will select schools (on a voluntary basis or following previous collaborations) and interact with stakeholders (regional and local school offices, school communities) to obtain their availability for participation. Attention will be given to contacting schools located in particularly disadvantaged areas and to include students with different sociodemographic and cultural backgrounds and levels of vulnerability. At each participating school, adolescents may participate in the different phases of the study.

The following inclusion criteria will be followed for participation in each included school: (1) having an age between 14 and 18 years, (2) having signed the consent form for the study participation (ie, consent to participate for adolescents less than 18 years signed by both parents—or legal representatives—or by the adolescent when 18 years); having sufficient knowledge of the Italian language.

### Procedure and Measures

#### Longitudinal 3-Waves Evaluation (Phase 1)

The longitudinal evaluation study (phase 1) will be articulated across 3-time points of assessment: baseline (T0: around months 3-5), at around 5 months (T1: months 8-10), and at around 10 months follow-up (T2: month 13-15). At each time point, a survey composed by a battery of self-reported questionnaires will be delivered through a dedicated web-based platform during regular school hours.

Diverse theoretical models of PSMU are operationalized in those battery of questionnaires (see Table 2 and Multimedia Appendix 1 for a detailed description of measures and questionnaires) to explore potential intra- and interpersonal psychosocial risk or protective factors of PSMU.

**Table 2:** Self-report questionnaires adopted in the SMART project^a^.

Role of the dimension in the longitudinal study ad questionnaire name			Investigated dimension		Questionnaire’s characteristics		Time
**Intrapersonal risk factors**							
	Difficulties in Emotion Regulation Scale-Short Form [33]	Emotion dysregulation		18 Items, 5-points Likert scale		T0, T1, and T2	
	Rosenberg Self-Esteem Scale [34]	Self-esteem		10 Items, 5-points Likert scale		T0, T1, and T2	
	Brief Self-Control Scale [35]	Self-control		13 Items, 5-points Likert scale		T0, T1, and T2	
	Satisfaction With Life Scale [36]	Satisfaction with life		5 Items, 7-points Likert scale		T0, T1, and T2	
	Strengths and Difficulties Questionnaire [37]	Adjustment difficulties and strengths		25 Items, 3-points Likert scale		T0, T1, and T2	
	Fear of Missing Out Scale [38]	Apprehension that others might be having rewarding experiences from which one is absent		10 Items, 5-points Likert scale		T0, T1, and T2	
**Interpersonal risk factors**							
	Inventory of Parent and Peer Attachment—Revised [39]	Attachment relationships toward mother, father, and peers		25 Items, 5-points Likert scale		T0	
	Preference for Online Social Interaction Scale (subscale of the Generalized Problematic Internet Use Scale 2) [40]	Preference for online social interaction		3 Items, 5-points Likert scale		T0, T1, and T2	
	Parental Phubbing Scale [41]	Perceived parental phubbing		9 Items, 5-points Likert scale		T0, T1, and T2	
**Primary outcome**							
	Bergen Social Media Addiction Scale [42]	Core addiction elements regarding SM^b^ use		6 Items, 5-points Likert scale		T0, T1, T2, and T3	
**Secondary outcomes**							
	Social Emotional Distress Scale—Secondary [43]	Emotional distress		10 Items, 4-point Likert scale		T0, T1, T2, and T3	
	Passive Social Networking Site Use Questionnaire [44]	Passive SM use		5 Items, 5-points Likert scale		T0, T1, T2, and T3	

^a^Time: time of administration; T0 (baseline) corresponds to months 3-5, T1 to months 8-10, and T2 to months 13-15. T3 is an additional time point (at the end of the app intervention) explained in phase 3.

^b^SM: social media.

Moreover, in a subsample of participants, momentary emotions or feelings (eg, happy, sad, tired, relaxed, nervous, quiet, each rated on a visual analogue scale) as well as the subjective use of SM during the past 2 hours will be recorded at 1 time point through an ad hoc app, using an ecological momentary assessment approach [45]. Momentary questions will be randomly delivered during nonschool hours (ie, from 3 to 10 PM), for a total of 4 times per day, for 2 weeks.

At baseline, 2 additional biological markers of PSMU will be randomly collected in vivo in a subsample of participants. First, the autonomic activity of the participants will be collected with the aim of testing whether a greater PMSU is associated with a decreased vagal (ie, parasympathetic) activity at rest. This autonomic parasympathetic activity—considered a physiological index of emotion dysregulation [24]—will be investigated through specific indices of heart rate variability (HRV) such as the high frequencies and the root mean square of successive differences between normal heartbeats [46]. The HRV will be continuously recorded during 10-minute resting condition using a wearable electrocardiogram device. The second biomarker is the presence of specific polymorphisms for serotonin gene transporter 5-HT1AR and 5-HT2AR that—according to the bipartite model of brain serotonin functions [25]—seem to be associated with a greater risk of developing internalizing disorders [47], such as PSMU. The presence of these polymorphisms will be tested through saliva samples, collected with a saliva stick and processed by the University of Perugia.

#### Qualitative Focus Group Study and Co-Design Approach (Phase 2)

Focus groups (FGs) have been widely used as a method for exploring people’s perspectives and experiences by using the group interaction to encourage the participants to explain, disagree, and share their views [48]. This method is particularly useful in research with adolescents [49]. Through FGs, we will explore the adolescents’ point of view regarding the following topics: the adolescents’ perspectives regarding the main purposes and modalities of SM use; the link between SM use, psychological distress, and well-being; risk factors associated with PSMU and protective strategies and resources that may be activated to promote functional use of SM. Each FG will be led by a moderator and an assistant (both with expertise in conducting FGs and in clinical psychology) and will last around 90-120 minutes each. Each FG will include around 8 to 10 adolescents, recruited through purposive sampling in the participating schools and balanced for sociodemographic variables, according to the suggestions of Palinkas et al [50], and the availability of the participating schools.

FGs will be conducted following a shared interview guide, including a set of open-ended questions and following a semistructured approach. While conducting the FGs, researchers will also pay attention to the group dynamics, ensuring that a good atmosphere is maintained. Before the FG, participants will fill out a brief questionnaire to collect background information (eg, sociodemographic information, emotional distress, and SM use) to describe the sample.

Co-designing of the app involves “a process of collective creativity” based on active collaboration between researchers, developers, and end users (ie, midadolescents) as “experts of their experiences” [31,51]. Originating from the field of participatory design, the co-design process allows to maximize engagement, satisfaction, and use of eHealth interventions for potential users [29]. General guidelines for the application of co-design methods and previous research experiences of the research team will be followed to organize and structure the co-design workshops [27,51,52], adopting agile design, design thinking, and user-centered design approaches (ie, cycles of design, develop, and test) [29]. The timeframe and duration of the workshops will be agreed with participating schools. Specifically, a “designing group” (ie, 2 researchers in clinical psychology, 1 computer engineer, and a subsample of midadolescents) will be established at the beginning of the second phase of the project. Members will be involved in a series of interactive workshops (organized in presence or through digital platform meetings) to define the main functionalities, features and contents of the app (alpha version) on the basis of: preexisting conceptual models on PSMU, existing evidence-based interventions for, preliminary evidence from the sample of Italian midadolescents, including their experiences, suggestions, and needs. During the workshops, the facilitators will encourage midadolescents to discuss the topic presented through practical techniques (eg, use of visual materials, scenario thinking, playful activities, participants’ interactions with prototypes and discussion on them) [53] for facilitating co-design while promoting meaningful engagement among adolescents [29]. Ideally, in the first workshop, midadolescents will be asked to discuss the general aims, functionalities, features, and contents of the self-help app, while the subsequent workshops will aim to collect inputs for adapting the alpha version to a beta version.

#### Feasibility and Pilot Study (Phase 3)

In phase 3, the co-designed self-help app will be delivered to all recruited midadolescents, who will be free to download the app on their personal iOS or Android device. For 1 month, participants will be invited to use the self-help app exploring the different contents and functionalities weekly, and to adopt these newly learned competencies in their daily life.

At the end of the self-help app use (T3; postintervention), feasibility will be investigated: (1) administering an adolescent-adapted version of the “Mobile App Rating Scale user version” (uMARS) [54], together with open-ended questions exploring the adolescents’ opinion about the app (eg, “Can you tell me about your experiences using the App?”; “What did you like most/least?”). The uMARS is a 20-item self-report measure of the perceived quality of apps, which includes 4 objective quality subscales (ie, engagement, functionality, aesthetics, and information quality) and 1 subjective quality subscale.

At this last time point (T3; around months 18-20), we will gather preliminary data on the effectiveness of the app intervention, administering the self-report questionnaires measuring the primary and secondary outcomes mentioned in phase 1. Given that the latter time point for the longitudinal study (ie, T2) falls a little before the start of phase 3, data on outcomes collected at that time point will be used as preintervention scores.

As a last step of this project, the app (eg, contents, features, and design) will be adapted on the basis of the emerging results.

### Sample Size Estimation

For the longitudinal study (phase 1) a convenient sample of 1216 adolescents will allow detection of a small effect size for regression-based models according to potential predictors (α=.05; power=0.80). After a 25% increase to account for potential dropouts, the estimated final number of participants will be 1520 adolescents.

For the HRV measure, to detect a medium effect size with 80% of power [55], at least 61 healthy adolescents need to be recruited. For the analysis of the genotyping serotonin transporter polymorphisms 5-HT1AR and 5-HT2AR, based on the effect sizes reported in Matsunaga et al [47], an a priori power analysis estimated that 191 participants would be necessary to detect a small-to-medium effect size (α=.05; power=0.80).

For phase 2, applying the criteria of data saturation, around 12 FGs distributed across schools in Italy are expected to be sufficient to explore all the relevant topics. The final number will be adjusted according to the data saturation criteria. As for the co-design workshops, a purposive sample of around 20 midadolescents is evaluated sufficient.

For phase 3, all the adolescents who took part in phase 1 of the project will be invited to participate in this phase. Considering that longitudinal studies on unguided self-help interventions report up to 63% of drop-out rates [56] and that this phase will start after several months from the first recruitment, we expect a final sample of around 400 adolescents participating in this phase, which is considered sufficient for feasibility and pilot aims.

### Intervention

The SMART app will aim to promote awareness and functional SM use. The app will use unguided web-based self-help approaches, that is a self-help program that does not include any contact with a therapist or researcher during its use, although it may include automatic reminders through app notifications (eg, to motivate the app use, or to complete an app session).

The app will be created according to the co-design described above and contents based on the more recent literature in the field, considering the evidence-based interventions already developed for PSMU and the empirical data derived from quantitative and qualitative data collected during the current project. To date, to the best of our knowledge, among the most widely used approach we have cognitive behavioral therapy, psychoeducational, positive psychology, and multifamily group therapy ones [57]. Among the preexisting theoretical models of PSMU, the systemic and cognitive-behavioral psychological models emerged. Specifically, the compensatory internet use theory [7] posits that PSMU could be conceptualized as a maladaptive coping mechanism that allows individuals to manage their negative emotions, to escape from disturbing issues and to regulate negative moods.

Although all the contents will be adapted according to the collected results and co-design, ideally the core features of the app will prioritize strategies aimed at improving emotional awareness, emotions regulation and interpersonal skills, specifically in relation to the context of SM use. Based on both theoretical and clinical knowledge and experience in the field, the app will ideally include several interactive modules, including: (1) an informative and awareness section with different educational contents (eg, information regarding SM use, consequences of PSMU, and SM role in defining individuals’ identity); and (2) a psychoeducational section aimed at promoting emotional awareness and skills and adolescents’ personal and interpersonal resources in order to prevent PSMU and promote functional SM use (eg, how to manage the pervasive apprehension that others might be experiencing rewarding activities that one could miss out; how to change negative thinking patterns; problem-solving strategies). Videos, practical scenarios, and examples (collected during FGs or co-created with adolescents during workshops) will be potentially used to promote engagement. The tone and contents of messages will be adapted according to the feedback from adolescents to ensure informative, nonjudgmental, and supportive communication as well as to motivate adolescents to use the app. Moreover, the app developers will use some specific tools to foster the readability and comprehensibility of the app (including, for example, the Simple Measure of Gobbledygook Index, Hemingway app).

### Ethical Considerations

In compliance with the ethical standards for research outlined in the Ethical Principles of Psychologists and Code of Conduct [58], approval by the Ethics’ Committee have been required and obtained by the Ethical Committee of the Umbria Region (protocol 4637/23). In order to participate in the study, written consent from parents or legal guardians and oral assent from midadolescents will be obtained. The informed consent will inform parents or legal guardians and midadolescents around the purposes of the research, the expected duration, the procedures, the absence of potential risks including physical, mental, and social injury, and participants’ right to withdraw at any time from the study if not willing to continue. Participation will be voluntary, and no monetary incentive will be given. In all the different phases of the study, data will be collected to assure participant’s privacy and confidentiality protection (eg, pseudonymization during the data collection and anonymization during the dissemination phase). All research teams will also protect the values, rights, and interests of the participants and respect basic ethical principles such as nonmaleficence, informed consent, and fair treatment. All data will be collected according to the EU General Data Protection Regulation.

### Statistical Analyses

Quantitative data will initially be screened for assumptions, including univariate and multivariate normality, and tested for the presence of outliers. Further, quantitative analyses will be complemented with effect sizes (which will be reported and interpreted according to guidelines) [59].

As for phase 1, we will test the longitudinal changes over time in PSMU as well as its psychosocial predictors, or the interactions between psychological distress (anxiety and depression) and PSMU, through 3-level mixed models (with repeated measures nested within participants, nested within classes) [60]. Ecological momentary assessment data will be examined through multilevel regression models, to test the interaction effects between SM use and emotional experiences over time. Finally, longitudinal profiles of participants will be examined through latent growth curve analyses [61]. Due to the relevant changes within adolescence, sensitivity analyses (based on sociodemographic variables, including age groups such as 14-15 years and 16-18 years) will also be carried out.

As for phase 2, each FG will be audio-recorded and transcribed verbatim. The transcribed text will be thematically analyzed using a 6-phase process for data engagement, coding, and themes development [62-64]: (1) familiarization with data and starting to create initial observations; (2) generation of initial codes; (3) searching for themes and discussion on how to combine and cluster different codes to form an overarching theme; (4) reviewing the themes checking them against the original data; (5) defining and naming themes; and (6) for each theme, representative quotes to contextualize and exemplify the themes will be selected to tell the reader a coherent story about the data. Analyses will be conducted by considering the recurrence but also meaningfulness as main criteria during the coding process. An inductive approach will be predominant [65,66]. However, to ensure that results will be meaningful to the research questions, in addition codes might be developed keeping in mind the main theoretical models of PSMU (such as the Multidimensional Model of Social Media Use [13], the Interaction of Person-Affect-Cognition-Execution model [67], and the pathway model [68]). To enhance rigor and trustworthiness of these procedures and analyses [69], and in accordance with Lincoln and Guba [70] credibility, transferability, dependability, and confirmability criteria, multiple strategies will be introduced. These will include prolonged engagement; persistent observation; 2 independent researchers will analyze the data separately; investigator triangulation by involving several researchers as research team members and presenting analysis at data group meetings; detailed descriptions of the research context to facilitate the assessment of its relevance and applicability to other contexts; purposeful sampling to ensure a diverse range of participants; reflexivity and researcher positionality; and maintaining an audit trail; and using transparent approaches to data analysis [69].

Finally, examples and scenarios will be stimulated during the discussion and might be used to inform the app (eg, collecting real-life examples of PSMU; situations in which negative experiences with SM are associated with well-being; protective strategies to PSMU).

In line with a mixed methods approach [71], quantitative and qualitative results will be synthesized and merged in order to achieve a better understating of the PSMU. Indeed, qualitative results will be used to deepen our understanding of the PSMU phenomenon, corroborating the findings from the quantitative design and/or adding potential different topics.

As for phase 3, feasibility (ie, uMARS mean scores and app usage) will be investigated through simple descriptives, examining whether participants met the thresholds for good feasibility: (1) mean uMARS subscale scores≥70% of the total will be considered indicative of good feasibility [72]; examining the app use (with ≥75% of participants with ≥10 app sessions considered indicative of a good feasibility) [72]. We will have a deeper understanding of the app feasibility by synthesizing the qualitative results. We will further examine whether the self-help app led to an actual change in primary and secondary outcomes through 3-level piecewise regression models, using the 4-time points from baseline (as defined in phase 1; T0) up to posttreatment (T3; M18-M20). To further strengthen these analyses, we will compute both a Reliable Change Index and a Clinically Significant Change for each participant [73,74]. These methods are used to test whether a change over time (in our case, from pre- to posttreatment) may be considered reliable (ie, taking into account the measure’s internal reliability) and clinically significant (eg, the posttreatment scores are within the boundaries of nonpathological scores).

Quantitative analyses will be performed using SPSS (version 29; IBM Corp), Hierarchical Linear Models (version 8.2; Scientific Software International, Inc), and MPLUS (version 8.4; Muthén & Muthén).

## Results

The project is funded by the Italian Ministry of University and Research as part of a national grant (PRIN, “Progetti di Rilevante Interesse Nazionale”), with the research team receiving an official notice of research funding approval for the current project in July 2023 (Project Code 2022LC4FT7). The study was preregistered on Open Science Framework, while the ethical approval was obtained in November 2023. We started the enrollments in December 2023, with the final follow-up data collected within May 2025.

As of July 2024, we completed 2 data collections (T0 and T1) and conducted the qualitative FGs of phase 2.

## Discussion

### Expected Findings

The innovative SMART project replies to crucial existing knowledge gaps on PSMU. First, to date, the conceptualization of PSMU is still debated. The SMART project emphasizes a person-centered approach since it underlines the need to focus not only on “how much” the SM is used, but also on “how” adolescents engage in this behavior. Second, a comprehensive evaluation of risk or protective factors for PSMU is still lacking. Thus, the potential effect of diverse variables at intra- and interpersonal psychological and biological levels, collected at several time points, is examined. Adopting complex longitudinal data analyses represents a third innovative aspect—as literature in the field relied mostly on cross-sectional designs—allowing us to test several hypotheses whose final aim is to disentangle the longitudinal associations between supposed risk or protective-factors, psychological distress, and the changes over time in PSMU. For example, the definition of different longitudinal profiles of primary or secondary PSMU outcomes will orient the scientific community about new research lines of inquiry and the development of tailored interventions. The adoption of EMA—a promising naturalistic and ecologic research method that provides longitudinally valid data and investigates SM use in real time—will additionally provide a fine-grained picture of adolescents’ experiences in their natural contexts, capturing both the variability over time in SM use, emotional experiences, and their interactive dynamic patterns. Third, an additional strength lies in the use of a mixed-methods design, combining quantitative and qualitative approaches to enrich the understanding of PSMU and of the relationships between the investigated variables.

In terms of impact, the project investigates a stage of life that has been strongly affected by the pandemic of COVID-19 [75]. The latter negatively impacted midadolescents’ need for autonomy and social interaction, causing anger and loneliness that some of them self-regulated through an increased use of SM. Therefore, the SMART project results may help better understand an actual adolescents’ need, worsened by the COVID-19 pandemic’s mental health burden. The SMART project invites adolescents to critically discuss SM use habits and needs and encourages them to share (eg, during FGs or co-design workshops) difficulties that adolescents might present as connected to SM use as well as the potential coping strategies adopted to face them. Even if this is not the aim of the FG, this may indirectly lead to a potential psychological benefit for adolescents in terms of normalization of the experience and reduction of isolation. The participatory approach actively engages adolescents to fully participate in designing an age- and culturally appropriate app-based self-help intervention. Empowering youths to play an active role in their well-being is one of the key foci of the participatory approach in promoting healthy communities [76].

Within the project, we will develop and evaluate an unguided self-help app, thus contributing to the advancement of knowledge on eHealth interventions for SM use and PSMU in adolescence. This simple and easy-to-use app, including interactive and supportive contents, will be based on adolescents’ preferences and inputs and on evidence-based intervention models, and will: (1) promote a healthier use of SM among midadolescents; (2) help them to become more aware of a potential PSMU; (3) mitigate the impact of psychosocial and biological risk factors on PSMU; and (4) boost their confidence through the use of more adaptive coping strategies while facing stressful events. The results from the pilot study may guide future multinational RCT studies for the evaluation of app effectiveness. Finally, in future research, the self-help app may be adapted to the special needs of adolescents with different PSMU profiles.

It is well known that untreated mental health problems, including psychological distress linked to PSMU-, have visible (eg, due to treatments) and invisible (eg, school dropouts, lower academic achievements) socioeconomic costs [77,78]. An unguided self-help intervention, such as SMART self-help app, may then be a cost-effective solution for promoting a better use of SM within this specific population, and which could be additionally included by schools and mental health professionals as a specific strategy in already existing interventions targeting psychological well-being of midadolescents.

Several dissemination activities maximize the impacts of the project, targeting the scientific community, schools, policy makers, and civil society. As regards schools, the project involves midadolescents and, potentially their caregivers and teachers, promoting interest around the PSMU. If, as suggested by some scholars [79], PSMU represents escapism from anxiety and depressed mood, informing, raising awareness, and sharing experiences about PSMU may have positive and substantial effects on psychological well-being, social exclusion, discrimination, educational difficulties, risk-taking behavior, and cyberbullying. Moreover, dissemination activities with caregivers (ie, organized at schools’ level at the end of the project) will be strategic, considering that provision of advice to parents has been scant and this kind of communication has not been specifically endorsed by governments or local communities. Dissemination events targeting policy makers will also contribute to informing policy recommendations on the apparent mismatch between perception of PSMU problems (by decision makers and adults in general) and real problems experienced by midadolescents. For example, attention has been primarily focused on digital safety (ie, cyberbullying, privacy issues) rather than on the psychological risk factors or impacts affecting adolescents and the promotion of functional SM use [80].

This study has some limitations. First, data will only be collected among Italian participants, thus our results may not be generalizable to other populations and cultures. Second, most of the measures used in this protocol are self-report, thus subjected, among the others, to response and social desirability biases [81]. The effectiveness of the SMART app will be tested through a nonrandomized design, thus limiting our possibility to make causal inferences. Finally, we have chosen to investigate only a fraction of the potential biopsychosocial predictors of PSMU among adolescents, thus we may have missed other important determinants of this problematic behavior.

### Conclusions

The SMART project contributes to the conceptualization of PSMU and deepens on its biopsychosocial antecedents among midadolescents, offering relevant scientific, technological, and socioeconomic impacts. The advancement of knowledge and the developed self-help app for PSMU responds to midadolescents’ increased loneliness and psychological burden due to COVID-19 pandemic and humanitarian crisis.

## Appendix

Multimedia Appendix 1 Table S1. Additional information about the self-report measures adopted in the SMART Project.

Multimedia Appendix 2 Peer-reviewer report from the Italian Ministry of University and Research (Ministero dell'Università e della Ricerca, MUR).

## Abbreviations

FG: focus group
HRV: heart rate variability
PSMU: problematic social media use
SM: social media
uMARS: Mobile App Rating Scale user version

## Data Availability

The data sets generated during this study will be available from the corresponding author on reasonable request.
