# Supplementary material for: Problematic Social Media Use Among Italian Midadolescents: Protocol and Rationale of the SMART Project
Source: JMIR Res Protoc. 2024 Sep 9;13:e58739. doi: 10.2196/58739 (PMC11420604; doi:10.2196/58739)
Supplement: Multimedia Appendix 1 [file resprot_v13i1e58739_app1.docx]

| **Questionnaire’s name (acronym)** | **Estimated Administration Time** | **Scoring** | **Reliability and Validity** |
| --- | --- | --- | --- |
| Difficulties in Emotion Regulation Scale-Short Form (DERS-SF) | 3 minutes | 6 subscales, total score | The DERS-SF demonstrated good internal consistencies, factor structure and validity (e.g., Kaufman et al., 2016). The scale was also validated in Italian (Rossi, Panzeri & Mannarini, 2023). |
| Rosenberg Self-Esteem Scale (RSES) | 1 minute | total score | The RSES evidenced good psychometric properties (e.g., Schmitt & Allik, 2005). An Italian validation is also available (Prezza, Trombaccia, & Armento, 1997). |
| Brief Self-Control Scale (BSCS) | 1 minute | total score | The BSCS showed a good internal consistency, factor structure and validity (e.g., Tangney et al., 2004; Manapat et al., 2021). This scale was also validated in Italian (Chiesi et al., 2020). |
| Satisfaction With Life Scale (SWL) | < 1 minute | total score | The SWL evidenced good psychometric properties, including internal consistency, internal structure and validity (e.g., Diener et al., 1985; Pavot & DIener, 2008). This scale was also validated in Italian (Di Fabio & Gori, 2016). |
| Strengths and Difficulties Questionnaire (SDQ) | 5 minutes | 5 subscales, 3 second-order factors | The SDQ evidenced poor to good internal consistencies, factor structure and validity (e.g., Goodman, 1997; Goodman, 2001). This scale was also validated in Italian (Essau et al., 2012). |
| Fear of Missing Out Scale (FoMO) | 1 minute | 2 subscales, total score | The FoMO scale showed good psychometric properties (e.g., Przybylski et al., 2013). This scale has been translated and validated in Italian (Casale & Fioravanti, 2020). |
| Inventory of Parent and Peer Attachment - Revised (IPPA-R) | 12 minutes | 3 subscales, total score | The IPPA-R evidenced good internal consistencies (e.g., Gullone & Robinson, 2005), factor structure and validity (Andretta et al., 2017). The scale was also validated in Italian (Pace, San Martini & Zavattini, 2011). |
| Preference for Online Social Interaction Scale (POSI subscale of the GPIUS-2) | < 1 minute | total score | The POSI subscale of the GPIUS-2 showed good psychometric properties (e.g., Caplan, 2010). The scale has been validated in Italian (Casale, Primi & Fioravanti, 2016). |
| Parental Phubbing Scale (PPS) | 2 minutes | 2 total scores (father & mother) | The PPS evidenced good internal consistencies, factor structure and validity (e.g., Pancani, Gerosa, Gui & Riva, 2021). The scale has been originally developed and tested in an Italian sample (Pancani, Gerosa, Gui & Riva, 2021). |
| Bergen Social Media Addiction Scale (BSMAS) | 1 minute | total score | The BSMAS showed a good internal consistency and overall psychometric properties (e.g., Andreassen et al., 2016; Zarate at al., 2023). The scale was also translated and validated in Italian (Monacis, De Palo, Griffiths & Sinatra, 2017). |
| Social Emotional Distress Scale – Secondary (SEDS-S) | 2 minutes | total score | The SEDS-S evidenced good internal consistency, factor structure and validity (e.g., Dowdy et al., 2018). The scale has not been validated in Italian yet. |
| Passive SNS Use Questionnaire (PSNSUQ) | < 1 minute | total score | The PSNSUQ showed good internal consistency (e.g., Chen et al., 2016). The scale has not been validated in Italian yet. |

**References**

Andreassen, C. S., Billieux, J., Griffiths, M. D., Kuss, D. J., Demetrovics, Z., Mazzoni, E., & Pallesen, S. (2016). The relationship between addictive use of social media and video games and symptoms of psychiatric disorders: A large-scale cross-sectional study. *Psychology of* *Addictive Behaviors*, 30(2), 252–262.

Andretta, J. R., McKay, M. T., Harvey, S. A., & Perry, J. L. (2017). Inventory of parent and peer attachment–revised scores in adolescents: A psychometric and person‐oriented study. *Family Relations*, 66(3), 527-540.

Casale, S., & Fioravanti, G. (2020). Factor structure and psychometric properties of the Italian version of the fear of missing out scale in emerging adults and adolescents. *Addictive Behaviors*, 102, 106179.

Casale, S., Primi, C., & Fioravanti, G. (2016). Generalized Problematic Internet Use Scale 2: update on the psychometric properties among Italian young adults. In *The Psychology of Social Networking. Identity and Relationships in Online Communities* Vol. 2 (pp. 202-216). De Gruyter Open.

Chen, W., Fan, C. Y., Liu, Q. X., Zhou, Z. K., & Xie, X. C. (2016). Passive social network site use and subjective well-being: A moderated mediation model. *Computers in Human Behavior,* 64, 507-514.

Chiesi, F., Bonacchi, A., Lau, C., Tosti, A. E., Marra, F., & Saklofske, D. H. (2020). Measuring self-control across gender, age, language, and clinical status: A validation study of the Italian version of the Brief Self-Control Scale (BSCS). *PloS One*, 15(8), e0237729.

Di Fabio, A., & Gori, A. (2016). Measuring adolescent life satisfaction: psychometric properties of the satisfaction with life scale in a sample of Italian adolescents and young adults. *Journal of Psychoeducational Assessment*, 34(5), 501-506.

Diener, E., Emmons, R.A., Larsen, R.J., & Griffin, S. (1985). The Satisfaction with Life Scale. *Journal of Personality Assessment*, 49, 71–75.

Dowdy, E., Furlong, M. J., Nylund-Gibson, K., Moore, S., & Moffa, K. (2018). Initial validation of the social emotional distress survey–secondary to support complete mental health screening. *Assessment for Effective Intervention*, 43(4), 241-248.

Essau, C. A., Olaya, B., Anastassiou‐Hadjicharalambous, X., Pauli, G., Gilvarry, C., Bray, D., ... & Ollendick, T. H. (2012). Psychometric properties of the Strength and Difficulties Questionnaire from five European countries. *International Journal of Methods in Psychiatric Research*, 21(3), 232-245.

Goodman, R. (1997). The Strengths and Difficulties Questionnaire: a research note. *Journal of child psychology and psychiatry*, 38(5), 581-586.

Goodman, R. (2001). Psychometric properties of the strengths and difficulties questionnaire. *Journal of the American Academy of Child & Adolescent Psychiatry*, 40(11), 1337-1345.

Gullone, E., & Robinson, K. (2005). The inventory of parent and peer attachment—Revised (IPPA‐R) for children: a psychometric investigation. *Clinical Psychology & Psychotherapy: An International Journal of Theory & Practice*, 12(1), 67-79.

Kaufman, E. A., Xia, M., Fosco, G., Yaptangco, M., Skidmore, C. R., & Crowell, S. E. (2016). The Difficulties in Emotion Regulation Scale Short Form (DERS-SF): Validation and replication in adolescent and adult samples. *Journal of Psychopathology and Behavioral Assessment,* 38, 443-455.

Manapat, P. D., Edwards, M. C., MacKinnon, D. P., Poldrack, R. A., & Marsch, L. A. (2021). A psychometric analysis of the Brief Self-Control Scale. *Assessment*, 28(2), 395-412.

Monacis, L., De Palo, V., Griffiths, M. D., & Sinatra, M. (2017). Social networking addiction, attachment style, and validation of the Italian version of the Bergen Social Media Addiction Scale. *Journal of Behavioral Addictions*, 6(2), 178-186.

Pace, C. S., San Martini, P., & Zavattini, G. C. (2011). The factor structure of the Inventory of Parent and Peer Attachment (IPPA): A survey of Italian adolescents. *Personality and Individual Differences*, 51(2), 83-88.

Pancani, L., Gerosa, T., Gui, M., & Riva, P. (2021). “Mom, dad, look at me”: The development of the Parental Phubbing Scale. *Journal of Social and Personal Relationships*, 38(2), 435-458.

Pavot, W., & Diener, E. (2008). The Satisfaction with Life Scale and the emerging construct of life satisfaction. *The Journal of Positive Psychology*, 3(2), 137–152.

Prezza, M., Trombaccia, F. R., & Armento, L. (1997). La scala dell'autostima di Rosenberg: Traduzione e validazione Italiana. Giunti Organizzazioni Speciali.

Przybylski, A. K., Murayama, K., DeHaan, C. R., & Gladwell, V. (2013). Motivational, emotional, and behavioral correlates of fear of missing out. *Computers in Human Behavior*, 29(4), 1841-1848.

Rossi, A.A., Panzeri, A. & Mannarini, S. (2023). The Italian Version of the Difficulties in Emotion Regulation Scale – Short Form (IT-DERS-SF): A Two-step Validation Study. *Journal of Psychopathology and Behavioral Assessment*, 45, 572–590.

Schmitt, D. P., & Allik, J. (2005). Simultaneous administration of the Rosenberg Self-Esteem Scale in 53 nations: exploring the universal and culture-specific features of global self-esteem. *Journal of Personality and Social Psychology*, 89(4), 623.

Tangney, J. P., Baumeister, R. F., & Boone, A. L. (2004). High Self-Control Predicts Good Adjustment, Less Pathology, Better Grades, and Interpersonal Success. *Journal of Personality*, 72(2), 271–324.

Wang, X., Gao, L., Yang, J., Zhao, F., & Wang, P. (2020). Parental phubbing and adolescents’ depressive symptoms: Self-esteem and perceived social support as moderators. *Journal of Youth and Adolescence*, 49, 427-437.

Zarate, D., Hobson, B. A., March, E., Griffiths, M. D., & Stavropoulos, V. (2023). Psychometric properties of the Bergen Social Media Addiction Scale: An analysis using item response theory. *Addictive Behaviors Reports*, 17, 100473.
