# Supplementary material for: Problematic Social Media Use Among Italian Midadolescents: Protocol and Rationale of the SMART Project
Source: JMIR Res Protoc. 2024 Sep 9;13:e58739. doi: 10.2196/58739 (PMC11420604; doi:10.2196/58739)
Supplement: Multimedia Appendix 2 [file resprot_v13i1e58739_app2.pdf]

Valutazione

Project code: **2022LC4FT7**

Project title:
Problematic Social Media use among Italian mid-Adolescents: from the identification of Risk/proTective factors to the co-design and evaluation of a self-help app. (SMART Project)

Coordinator: **DELVECCHIO Elisa** ERC: **SH4_3**

University: **Università degli Studi di PERUGIA**

Evaluation Summary Report

1. Quality of the research project - scientific merit and innovative nature of the project from an international perspective

CRITERIA	EVALUATION
a) Clarity and originality of the project objectives	*(provide a score from 1 to 10)* **10**
b) Relevance of the proposed project as to the specific scientific area	*(provide a score from 1 to 10)* **9**
c) Coherence as to the methodology adopted in terms of the project structure and the relative objectives with specific reference as to the contribution of the local units	*(provide a score from 1 to 10)* **9**
d) Positioning of the project as to the state of the art in the specific scientific area	*(provide a score from 1 to 10)* **9**

1. The project objectives are structured and presented clearly and very convincingly. 2. Project has three interconnected main aims: (i) To advance knowledge and understanding of problematic use of social media by adolescents; (ii) to co-design a self-help app to enable greater awareness social media use and its various functions; (iii) to test this prototype app for its effectiveness with a view to to improve and adapt later version. 3. The project has sets out its methodology clearly by proposing five discreet work packages carrying out research and delivery from across Italy. 4. This is an ambitious butvery timely project which help us both understand a pressing social issue and also addressing it through the digital technology that is responsible for the problematic engagements with social media.

With reference to the score awarded, please provide the relative motivations by answering tothe following questions:

1. *To what extent are the project objectives structured in a clear and original manner?*
2. *What are the key points of the proposed project as to the specific scientific area?*
3. *In what way are the adopted project methodology and objectives, with specific reference as to the contribution of the local units, coherent with the project goals?*
4. *To what extent do the objectives go beyond the state of the art in the specific scientific area?*

2. Composition of the research team, feasibility and appropriateness of the project – scientific merit of the research team, feasibility of the work plan and appropriateness of the funding request

CRITERIA	EVALUATION
a) Expertise of the Principal Investigator, the heads of the local units and the research team	*(provide a score from 1 to 10)* **9**
b) Ability as to the implementation of the proposed project (qualification, composition and complementarity of the team)	*(provide a score from 1 to 10)* **10**
c) Organisation of the project as to the proposed objectives, the timeframe considered necessary to complete the project and the resources required (consumables, equipment, management)	*(provide a score from 1 to 10)* **10**
d) Consistency of the time commitments of the members of the research team, appropriateness and relevance of the spending plan as to the objectives and time distribution of the activities	*(provide a score from 1 to 10)* **9**

1. The PI and the team members have an excellent record for their stage of career. 2. The project is exceptionally well designed at a level of detail suitable for submission to a large scale appropriate Horizon Europe call for funding. This high level of attention to detail provides strong evidence both for its coherence and feasibility. 3. The project aims to deliver significant research results as well as a potentially very useful app for comparing a serious societal and psychological problem afflicting adolescents. 4. The time commitment of the team members is consistent with the demands of the project. The spending plan is coherent.

With reference to the score awarded , please provide the relative motivations by answering to the following questions:

1. *To what extent have the PI, the local unit heads and the research team demonstrated their scientific expertise?*
2. *To what extent is the project feasible and coherent in terms of competence, composition, and complementarity of the research team?*
3. *To what extent is the project consistent with the proposed objectives, the timeframe considered necessary for its completion and the resources required (consumables, equipment, management)?*
4. *To what extent is the time commitment of the members of the research team consistent with the objectives and activities of the project? To what extent is the spending plan coherent and relevant to the objectives and activities of the project)?*

3.Project Impact

CRITERIA	EVALUATION
Assessed on the basis of one or more of the following criteria:<br>• advancement of knowledge<br>• technological innovation and/or industrial applications<br>• compliance with the principle of Do Not Significant Harm (DNSH)<br>• scientific community and its strengthening<br>• internationalisation of Italian research<br>• social welfare and/or cultural developmen<br>• dissemination/sharing of scientific knowledge	*(provide a score from 1 to 20)* **20**

I think this is an outstanding project of its kind. It is designed with great care to detail and promises innovative research outcomes as well as a practical application. Dissemination of the results have also been planned meticulously though all the phases of the project. The are plans for both research and application outputs for various target groups, including the scientific community, civil society, and policy makers. The project, once fully implemented, will contribute to the social welfare of adolescents, their families and teachers. An app to help to reduce problematic use of social media can have application in all countries where such behaviour is common place. So, the project can help to internationalise Italian research once it is adapted to other linguistic and cultural contexts.

As to the awarded scoring, kindly provide the relative motivations by answering, also aggregately, the following questions:

1. *Does the project advance knowledge?*
2. *Does the project measure up to the challenges that research faces in terms of technological innovation and industrial applications?*
3. *Does the project respect the principle of Do Not Significant Harm (DNSH)?*
4. *Will the project have an impact on the scientific community? How will it strengthen it?*
5. *Will the project increase the internationalisation of Italian research?*
6. *Will the project contribute to social welfare and/or cultural development?*
7. *Does the project propose actions to disseminate knowledge and its results?*

TOTAL SCORE **95**

© 2023 - MUR - Direzione Generale della Ricerca - in collaborazione con CINECA
